# Simulation Study of Chain-like Body Translocation through Conical Pores in Thick Membranes

**DOI:** 10.3390/membranes12020138

**Published:** 2022-01-24

**Authors:** Zbigniew Domański, Andrzej Z. Grzybowski

**Affiliations:** Department of Mathematics, Czestochowa University of Technology, PL-42201 Czestochowa, Poland; andrzej.grzybowski@pcz.pl

**Keywords:** conical pore, solid-state nanopore, statistics, thick membrane, translocation

## Abstract

Artificial membranes with conical pores and controllable thickness reveal ionic-transport capabilities that are superior compared with those offered by cylindrical pores. By simulating the translocation of an abstract chain-like body through a conical pore in a membrane with a variable thickness, we formulate a statistical model of the translocation time τ. Our rough model encodes the biochemical details of a given real chain-like molecule as evolving sequences of the allowed chain-like body’s conformations. In our simulation experiments, we focus primarily on pore geometry and kinetic aspects of the translocation process. We study the impact of the membrane thickness *L*, and both conical-pore diameters $\phi_{cis},\phi_{trans}$ on the probability distribution of τ. We have found that for all considered simulation setups, the randomness of τ is accurately described by the family of Moyal distributions while its expected value $\langle \tau \rangle$ is proportional to $L^{\xi }$, with ξ being dependent on $\phi_{cis},\phi_{trans}$.

## 1. Introduction

A living cell, protected from the environment by a tiny membrane [1], fulfills actions required by the organism to which it belongs due to an orchestrated transport of molecules through membrane pores [2]. Although the protective role played by membranes is the most crucial in nature, it is not, however, unique [3]. Natural and artificial membranes are employed in life sciences, biochemistry and a variety of industrial processes [4,5]. Examples are membranes functioning as parts of filtering systems that clean fluids from suspended undesired particles [6,7,8,9]. Depending on particular needs, membranes have to provide and preserve desired transportation capabilities [4,10] and service quality [11,12].

During a chain-molecule transfer along a pore residing inside of a given membrane, the molecule and pore interact [13,14,15,16]. The measurable characteristics of such interaction enable researchers to track information about the molecule [17,18]. From this point of view, pores have grown into instruments, offering a wide spectrum of applications. Today, membrane pores are situated at the centre of a technique called nanopore force spectroscopy [13], i.e., they are engaged in detecting, identifying and sorting small molecules [9,19], including the sensing of a single molecule [20].

Two classes of engineered nanopores are applied as tools in biotechnology: (i) naturally occurring biological nanopores [19] and (ii) solid-state, artificial ones [21,22]. As biological pores are assembled with the atomic precision and tuned through genetic modifications, they are very small and sensitive to environmental changes, which may trigger problems with their structural stabilities [13]. Solid-state nanopores are essentially resistant to their environment, mechanically durable and admit tuning with sub-nanometric precision [12].

When dealing with artificial membranes that are expected to offer highly efficient transport properties, pore shape [16,23,24,25,26] and membrane thickness [27,28,29,30,31] are the main geometrical factors that should be carefully tuned in order to facilitate chain-bodies’ passage, prevent pore clogging [32] and guarantee the desired throughput [33]. The reported results of experiments show that: (i) conical pores strongly enhance the rate of transport [12,16], and (ii) the translocation of ions through a synthetic membrane is affected by the membrane’s thickness [30,31]. It is worth mentioning that among a multitude of theoretical studies devoted to polymer passage through complex geometries, only a few papers concentrate on conically-shaped pores, e.g., [34,35,36,37]. Interestingly, the conical shape proves to also be beneficial with respect to the shape of an electric field assisting in the field-driven passage of biomolecules along an insulating solid-state nanopore [33].

The present paper focuses on a chain-like-body translocation efficiency. We analyse, statistically, how this efficiency changes when a cylindrical pore in a given thick membrane is replaced by a conical one. The required data are collected from simulations carried out with a sequential algorithm that enables us to mimic a chain-like-body transport along a pore. Although our algorithm can be tuned in accordance with conditions imposed by chemical constituents of both a chain-like-body and pore, in this study we employ its bare version. The chain is viewed as an unbreakable sequence of self-avoiding segments and the pore and membrane are treated as rigid structures. A small bias, with respect to the direction of the random movement of the chain’s segments, is imposed inside the pore as the only factor discriminating between the cis and trans sides of the membrane.

Our goal is to capture the most basic relations between conical pore diameters ($\phi_{cis},\phi_{trans}$), membrane thickness (*L*) and translocation time (τ). Through extensive numerical simulations, we have explored a discrete set of representative values of conical pore diameters jointly with membrane thicknesses in order to quantify characteristics of the translocation process. The main results of our numerical experiment refer to: (i) probability distributions ψ of translocation times τ and (ii) mean $\langle \tau \rangle$. Based on the simulation results, we argue that ψ(τ) are accurately approximated by the family of Moyal distributions, whereas $\langle \tau \rangle$ and the membrane thickness (*L*) are related through a power law function whose exponent depends on pore diameters ($\phi_{cis},\phi_{trans}$).

In Section 2, we specify the model and computational framework. Section 3 provides the presentation and discussion of the simulation results. Finally, in Section 4, we summarise our findings and formulate conclusions.

## 2. Model Description and Computational Framework

This section specifies our model of a chain-like body (CLB) together with the algorithm that enables us to mimic a chain-like molecule’s motion. Our aim is to probe the extent of time (τ) that the CLB spends in a given pore when traversing a thick membrane. Specifically, we compare τ required by the CLB to pass through a conical pore to the time necessary to translocate along a cylindrical pore by the same CLB.

### The Sequential Algorithm and Monte Carlo Experiments

Numerous CLB-related research problems have been reported in the literature over the last decades. Apart from biophysics and chemistry experiments, we are also presented with a large number of studies involving computer simulation experiments. Computational schemes used in the field of CLB dynamics generally fall into two classes: (i) molecular dynamics (MD) and (ii) Monte Carlo (MC) simulations. Within the MD simulations, an appropriate second-order differential equation of motion is developed and integrated over time by some numerical methods [34,37]. In the MC approach, a set of probabilistic rules for the stochastic generation of a sequence of CLB configurations is used to trace the system trajectory in the configuration space. The former group of simulations is designed primarily to model the configurations’ changes occurring locally, on the scale of a bond. The latter one, in turn, is addressed to the global properties of the chain conformations when some large-scale characteristics of the chain’s movements effectively become independent of chemical details. This means that the chains can be mapped on coarse-grained models and investigated via computer simulations—a principal goal of our study.

Algorithms that are frequently employed in the MC simulations are presented and discussed in various papers and/or monographs, see e.g., [38,39]. Among the most popular algorithms, we find the self-avoiding walk (SAW), the bond–fluctuation model (BFM), the slithering snake (SS) algorithm, the extended reptation algorithm (eRA), Rosenbluth–Rosenbluth method (RR) and the pivot algorithm for the SAW. All these algorithms have specific advantages and disadvantages that are reported in the literature. For instance, algorithms that are based on local moves are too slow for systems with large numbers of segments while those enabling non-local CLB updates (such as the PA) frequently produce unacceptable configurations.

In our studies, we use a sequential algorithm (SA) that was introduced in [40,41]. When comparing the SA to the above algorithms in the context of their structural similarities and/or differences, one can observe that the SA comprises advantages of local and non-local moves as well as a preliminary search of actually available configurations. It can also be shown that by an appropriate choice of parameters we can simulate all the above mentioned algorithms as the special cases of the SA. On the other hand, the SA includes a built-in tension propagation mechanism and varying bond length.

A detailed description of the SA can be found in [40,41]. However, for the reader’s convenience, we repeat the most important parts of the description of the algorithm in the section Supplementary Materials. In this Subsection, we only briefly introduce those notions and facts necessary for understanding the later-presented results.

An abstract position of the CLB is a finite sequence of points ${\left\{c_{i}\right\}}_{1\leq i\leq N}$ located in nodes of a 2D square lattice with the lattice constant a=1. This means that $c_{i}$ are points with integer coordinates, and the distance (*Dis*) between any pair of its consecutive elements is bounded by given limits:

(1) $$\Delta_{min}\leq Dis\left(c_{i},c_{i+1}\right)\leq \Delta_{max}$$

The elements, $c_{i}$, of such a CLB sequence are called segments, and their number, *N*, is the length of the CLB. In our experiments $\Delta_{min}=2$ and $\Delta_{max}=4$, meaning that the shortest possible bond consists of two segments and one empty node between them (obviously the longest possible bond contains three empty nodes). We will refer to this fact later on while discussing the simulation results.

Within our algorithm, any change of location, called a move, from one position of the CLB to another is achieved via random allowable steps made by its segments [40]. By making a step the segment may create tension in the CLB structure. Particularly, it may happen when the distance between consecutive monomers does not satisfy the condition (1). In such circumstances, consecutive segments make steps as long as the tension is sustained in the structure. If the first step does not create any tension in the CLB structure, then the move may consist of a single step only. Consequently, every move of the CLB is initialized by a step of only one randomly selected segment and then its step is followed by a number of steps of subsequent segments, reflecting the tension propagation through the CLB. Any driving force [42,43] or other environmental [13] impacts on the CLB can be incorporated into the simulation experiments by proper tuning of a bias on the probable directions of random translocations of the segments, as well as on the choice of the segment initiating the move. In the considered case, a small such bias was imposed to drag the CLB segments along a pore between *cis* and *trans* sides of the membrane. Moreover, while selecting a segment that initiates a single move, the ones inside the pore were slightly more probable.

**Figure 1:**
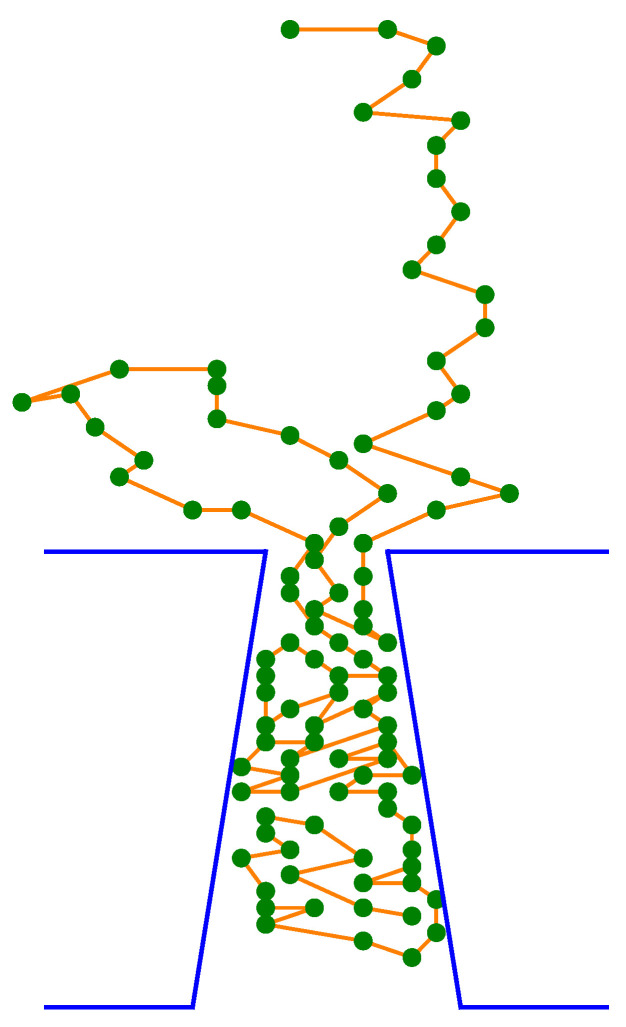
A schematic view of a chain-like body when passing through a conical pore.

In the numerical experiments presented in this paper, we study the translocation of a CLB through a conical pore in a thick membrane, see Figure 1. In our simulation model the pore geometry is characterised by the following quantities: the inlet and outlet diameters, $\phi_{cis}$ and $\phi_{trans}$, respectively, and the pore length *L*.

We focus on the analysis of the impact of $\phi_{cis}$, $\phi_{trans}$ and *L* on translocation time probabilistic characteristics.

## 3. Results and Discussion

We have carried out extensive simulations of the model specified in Section 2 to gather data sets that are required by reliable statistics of translocation times. Specifically, in all simulations we have employed a simple sample comprised of $M=10^{3}$ initial configurations of an *N*-segment CLB, each with the first segment placed in the entry of the pore. We have collected data for different arrangements of values of $\phi_{cis},\phi_{trans}$ by varying pore length, *L*, while keeping a predetermined number, *N*, of the CLB segments. Specifically, in our Monte Carlo experiments, the inlet and outlet diameters, $\phi_{cis}$ and $\phi_{trans}$, respectively, assume values from the set $\left\{3,4,6,8,12\right\}$, whose elements are expressed as the number of available nodes. More precisely, any combination of these diameters’ values are taken into account. *L* varies within the set $\left\{5,10,15,20,25,50,75,100\right\}$, representing the numbers of available nodes, while the CLB consists of N=100 segments.

When a CLB enters a pore its configuration is determined by the *cis* side of the membrane. In order to initiate the translocation process, the CLB has to be captured by the pore. During the early stages of translocation, however, it is frequently observed that the CLB retracts from the pore. Due to that, multiple variants of electrostatic focusing [44,45] are applied in the experiments to increase the rate at which polymers arrive and thread into the channels.

**Figure 2:**
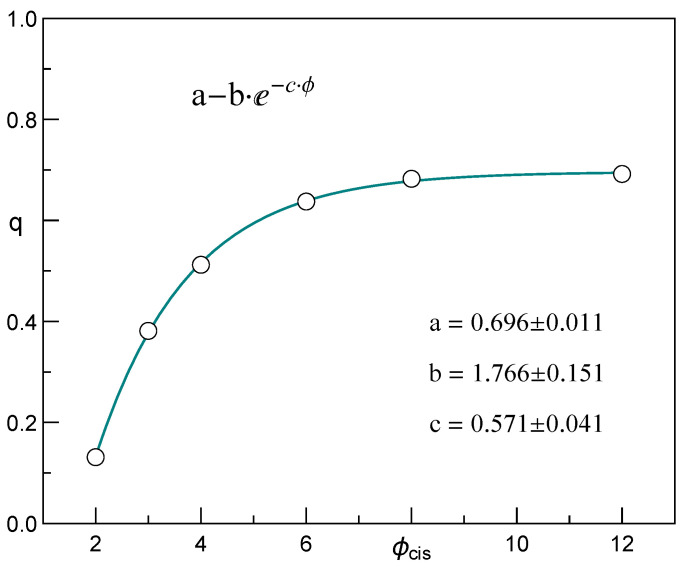
Fraction *q* of successful capture events: open circles—data points; green line—the best fit. The errors indicate 95% confidence intervals.

A smart choice of initial bio-molecule position also increases the fraction of successful capture events [13]. In our simulations, each CLB started from a configuration with the CLB head exclusively placed in the pore opening. We have counted every CLB retraction and were thus able to compute the resulting fraction *q* of successful capturing followed by the threading of the CLB through a pore. In Figure 2 we display empirical values of *q* along with the corresponding best fit.

### 3.1. Probability Distribution of Translocation Time

We start with a search for the probability distribution that best approximates the probability law that governs the randomness of the translocation time. For this purpose, we have thoroughly examined all gathered data sets by applying a range of suitable goodness-of-fit tests, among others, the Cramer–von Mises and Anderson–Darling ones [46]. Based on the results of those tests, we have found that the family of Moyal probability distributions [47] provides the best fit to the collected empirical values of τ for all examined setups of the geometric parameters of the conical pore. This conclusion extends our previous observations related to a thin membrane [40] or a cylindrical pore [48]. The most popular (and most elegant) form of the Moyal probability density function is as follows:

(2) $$\psi \left(\tau \right)=\frac{1}{s\sqrt{2\pi }}\exp\left[-\frac{1}{2}\exp\left(-\frac{\tau -m}{s}\right)-\frac{\tau -m}{2s}\right],$$

where *m* is the peak’s ordinate and *s* is a scale parameter of the Moyal pdf.

**Figure 3:**
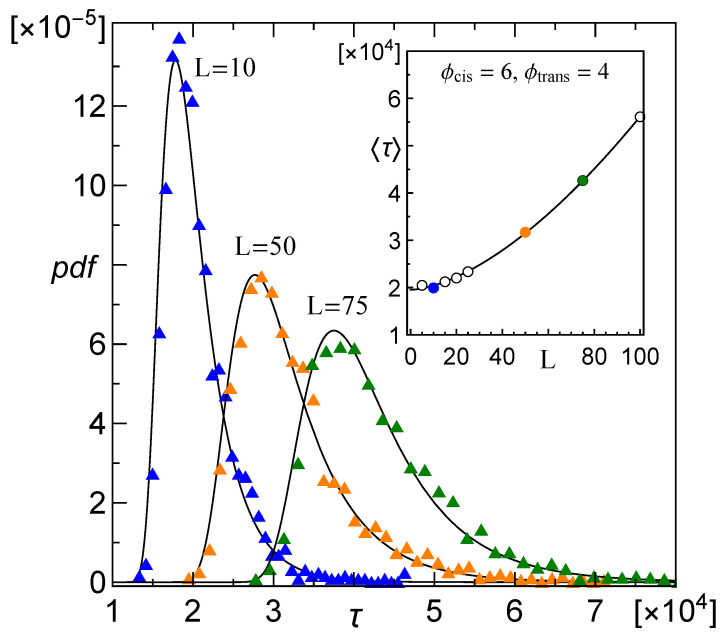
Empirical distributions of translocation time, τ, of a CLB with N=100 segments computed for increasing membrane thickness, *L*, and pore diameters $\phi_{cis}>\phi_{trans}$. Solid lines follow Equation (2). The inset shows respective mean values $\langle \tau \rangle$. The sample size is $10^{3}$.

The Moyal distribution may also be parameterized directly by its expected value $\langle \tau \rangle$ and standard deviation σ. In such a case, in the above formula, the parameters *m* and *s* should be replaced with the following linear expressions

(3) $$\begin{array}{ccc} m & = & \langle \tau \rangle -\frac{\sigma \sqrt{2}}{\pi }\cdot \left(\ln2+\gamma \right), \end{array}$$

(4) $$\begin{array}{ccc} s & = & \frac{\sigma \sqrt{2}}{\pi },\;\;\;\;\;\; \end{array}$$

where γ≈0.577 denotes Euler’s constant. As it is much more convenient to study the behaviour of the distribution moments than of the parameters m,s, in the remaining parts of the paper we will refer to the $\langle \tau \rangle$ and σ as the Moyal distribution parameters. In Figure 3 we display empirical distributions of translocation time and their best fits given by Equation (2) with parameters computed from collected data. It is worth mentioning that the Moyal pdf and its inverse, the Lamber-W function, take part in models that refer to a variety of biophysical systems [49,50].

### 3.2. Dependence of the Mean Translocation Time on the Membrane Thickness

**Figure 4:**
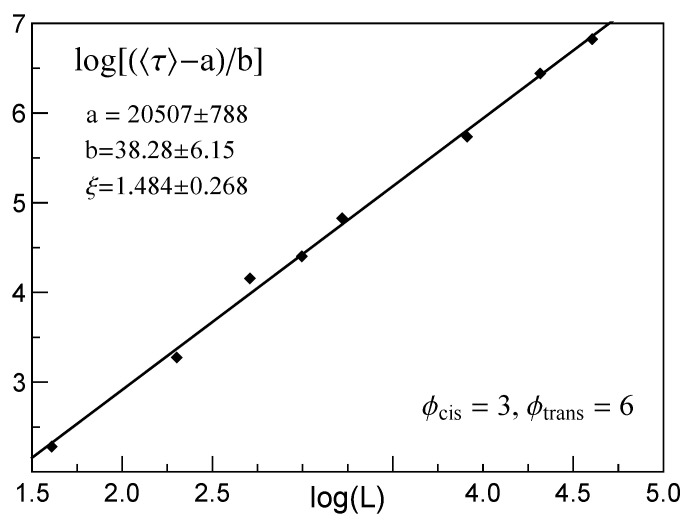
Illustration of the power law relation given by Equation (5).

In accordance with the results of our numerical experiment, we observe that a power law relation

(5) $$\langle \tau \rangle =a+b\cdot L^{\xi }$$

best fits the mean time, $\langle \tau \rangle$, spent by CLBs when they passed pores in membranes with growing thicknesses of *L*. The parameters a,b and the index ξ depend on $\phi_{cis}$ and $\phi_{trans}$. An illustrative example of relation (5) is presented in Figure 4.

**Figure 5:**
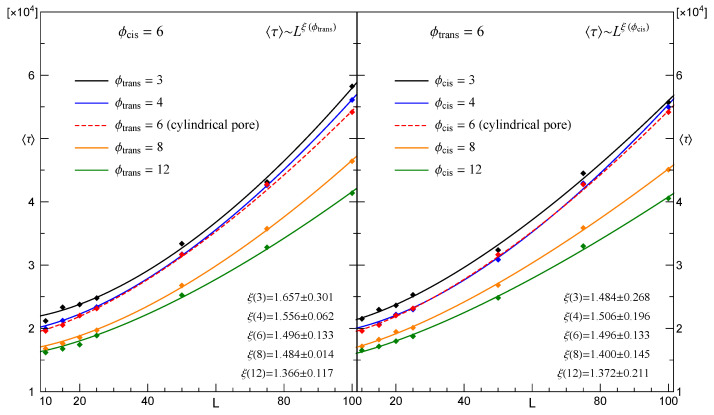
Empirical mean translocation time $\langle \tau \rangle$ vs. membrane thickness *L* for a CLB passing through conical pores. **Left** panel: $\phi_{cis}=\mathrm{const}.$; **right** panel: $\phi_{trans}=$ const. Lines are given by Equation (5) with parameters estimated from the data.

More detailed information about the relations between $\langle \tau \rangle ,L,\phi_{cis},\phi_{trans}$ is provided in Figure 5 along with that referring to corresponding cylindrical pores.

When comparing the mean translocation times displayed in Figure 5 for different arrangements of pore diameters, it is clearly seen that $\langle \tau \rangle$ differs only slightly when the direction of translocation is reversed. Obviously, such a difference will grow if membrane thickness increases. This is because $\langle \tau \rangle$ and *L* are related through Equation (5) and the estimated values of the corresponding exponents, ξ, differ; see the data displayed in the left and right panels. When comparing values of ξ for a given pair of $\phi_{cis},\phi_{trans}$, it is seen that $\langle \tau \rangle$ referring to $\phi_{cis}<\phi_{trans}$ is smaller than that related to $\phi_{cis}>\phi_{trans}$. Such a slowing-down appears when $\phi_{cis}>\phi_{trans}$, because then the average speed of the segments that leave the pore is less than that at which the remaining segments enter the opening, so the segments tend to get grouped inside the pore.

**Figure 6:**
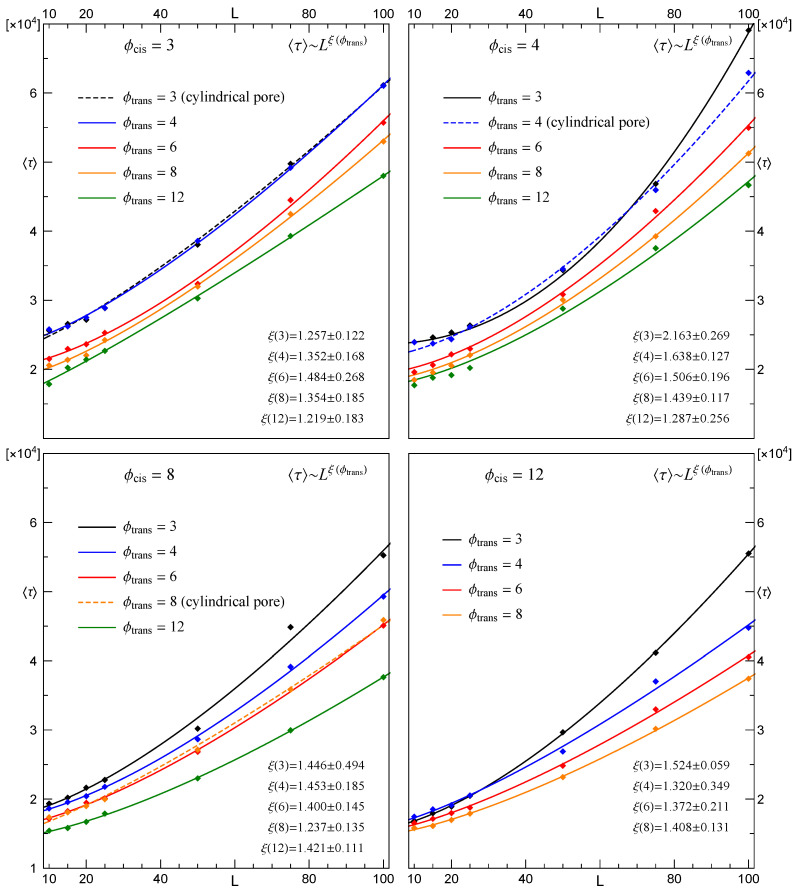
The same as in Figure 4. Each panel corresponds to $\phi_{cis}=\mathrm{const}.$ and growing $\phi_{trans}$.

Translocation times through pores with other diameters than those presented in Figure 5 were also examined, and the respective results are shown in Figure 6. An obvious feature concerns a shortening (lengthening) of translocation time due to an increasing (decreasing) difference between values of $\phi_{cis}$ and $\phi_{trans}$.

**Figure 7:**
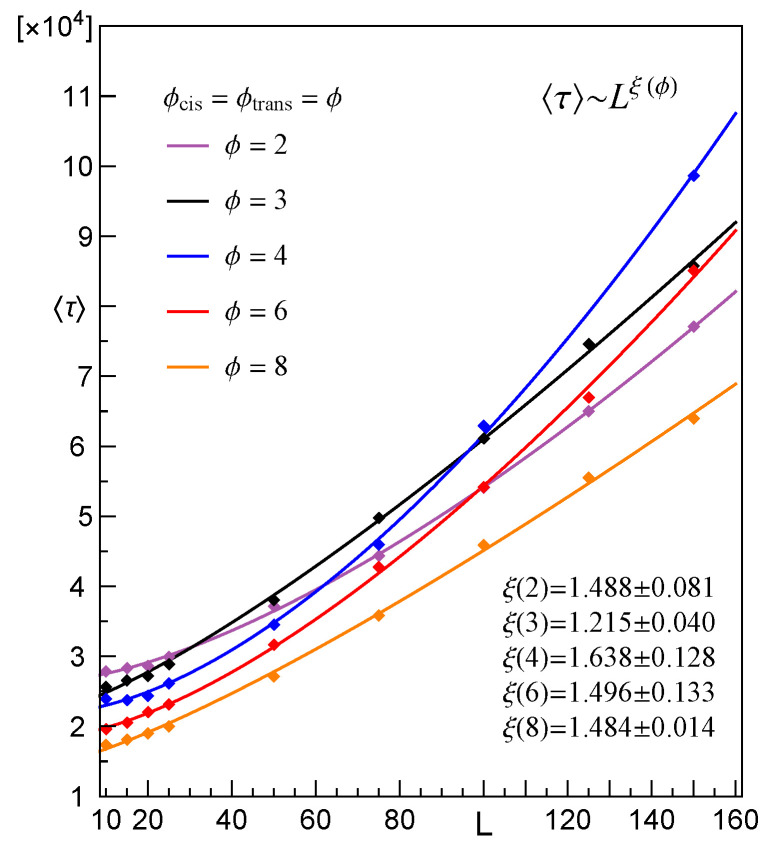
Empirical mean translocation time $\langle \tau \rangle$ vs. membrane thickness *L* for CLB passing through cylindrical pores with exemplary values of ϕ.

Additionally, Figure 7 displays $\langle \tau \rangle$ corresponding to the CLB translocations through cylindrical pores of length L≤150. A part of this data was displayed in Figure 5 and Figure 6 as data points and respective dashed lines.

A more in-depth analysis of how and to what extent pore diameters affect $\langle \tau \rangle$, in reference to membrane thicknesses, is presented in the next section.

### 3.3. The Regression Models for the Translocation–Time-Distribution Parameters in Relation to Pore Diameter

In this part of the paper, we present the regression models relating the pore length *L* and the diameters $\phi_{cis},\phi_{trans}$ with the Moyal distribution parameters $\langle \tau \rangle ,\sigma$. These particular models presented here are developed based on the data obtained for the CLB consisting of N = 100 segments. In our research, however, qualitatively similar results have been observed for other lengths of the CLBs.

We start with the models for the expected value $\langle \tau \rangle$. In the model-building process, we have taken into account various potentially interesting and naturally interpretable shapes of the functional dependency between $\langle \tau \rangle$ and the above pore-parameters. It turns out, on the basis of the analysis of the models’ quality characteristics, that the best-estimated model function is the following:

(6) $$\langle \tau \rangle =b_{0}+b_{1}\phi_{cis}+b_{2}\phi_{trans}+b_{3}\frac{\phi_{cis}}{\phi_{trans}}$$

with $b_{i}$’s being the estimates of the regression coefficients. Their values along with some important model quality characteristics are presented in Table 1. The symbol $R^{2}$, as usual, denotes the coefficient of determination, i.e., the most popular goodness-of-fit statistic. Other presented characteristics of the model quality are the so-called model standard error (MSE) and the coefficient of variation (CoV) i.e., the well-known measure of relative dispersion of the observations around the model response surface. Apart from these values, we also present the *p*-values related to particular estimates, $b_{i}$—they are placed below the estimates’ values. Roughly speaking, they tell us what the probability is that a given/related explanatory variable is unnecessary in the model.

First, let us note that the developed models, being quite simple and natural, manifest very good compatibility with the data—the coefficients of determination are high; in all cases, above 83%. Moreover, both the quality characteristics MSE and CoV also indicate good quality and high reliability of the models, especially CoV, which amounts only to about 5%.

Looking at the regression coefficients of the models provided in Table 1, we can instantly see that they confirm the expected relation: the bigger the openings, the shorter time of the CLB translocation, see Figure 5.

However, a slightly deeper analysis of the results also reveals a much less obvious fact. Let us look at the provided *p* values. We see that the ones associated with the estimates $b_{0},b_{1},b_{2}$ are very small, indicating that the related explanatory variables (i.e., the intercept, $\phi_{cis}$, and $\phi_{trans}$) are undoubtedly very significant in all these models. Now let us focus on the coefficient $b_{3}$, which reflects the impact of the ratio $\frac{\phi_{cis}}{\phi_{trans}}$ on the passage time. We see that for relatively thin membranes (L=10,25) the *p*-values are very high (0.3951,0.7656), whilst for the thicker ones the *p*-values are really very small (0.0299,0.0015,0.0062). At this point, we should note that, from the standpoint of the art of regression model building, for thin membranes the last component should be removed from the models (6), whilst for thick membranes, only this component is particularly important. This is really interesting because it tells us that for thin membranes, the diameters $\phi_{cis},\phi_{trans}$ affect the translocation time independently, i.e., the impact of the change of one of these diameters does not significantly depend on the actual size of the other diameter. In contrast, for thick membranes we observe the interaction effect—the impact (on the translocation time) of the size-change of one diameter depends on the size of the other opening.

The possible explanation for such an observation is that for longer pores the chance of segments clustering increases, especially when $\phi_{cis}>\phi_{trans}$. Obviously, each cluster of segments slows down the whole CLB-translocation process, and the thicker the membrane, the more clusters can be created inside the pore.

**Figure 8:**
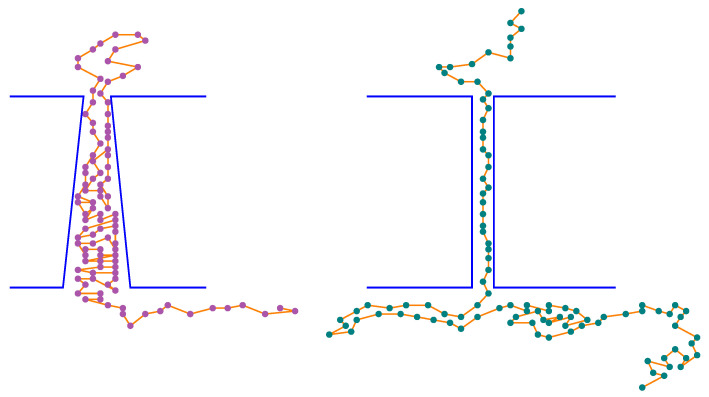
A schematic view of CLB translocations along narrow pores: conical vs. cylindrical.

At this point, it is a good moment to report on yet another observation that is related to the previous one. This remark refers to the case where $\phi_{trans}=2$. In our research, this value of the outlet diameter was also taken into account. However, it turned out that for L>20, within about 10% of all such cases, the CLB gets stuck in the pore for good (we had to stop the simulations for computation time reasons). Moreover, in the remaining (i.e. successful) transitions, the passage time varied dramatically; from several thousands to millions of steps, indicating that very often the CLB was “almost stuck” in the pore. It is worth mentioning, here, that for the cylindrical pores with identical diameters $\left(\phi_{cis}=\phi_{trans}=\phi =2\right)$ the translocation time is the shortest one, when comparing with $\langle \tau \rangle$, which refers to pores with 2<ϕ<8 and L>100, see Figure 7 and for other details refer to [48]. We see that the conical geometry of the pore is very significant in these cases. Recall that $\phi_{trans}=2$ means that only two lattice-nodes are available for CLB segments while passing this opening. However, one of the assumed restrictions imposed on the CLB structures is $\Delta_{min}=2$, meaning that the shortest possible bond needs 3 available nodes. This fact can be the possible reason for such behaviors of the CLB while passing through the pore—if the pore is cylindrical, then no clustering of the CLB segments is possible, because the diameter is too small. Hence, the CLB moves always in a “head-to-tail” fashion. On the other hand, in the conical pores, if the diameter $\phi_{trans}=2$ then the tempo of the segments that leave the pore is too small in comparison to the speed at which the further segments enter the opening with $\phi_{cis}\geq 3$. As a consequence, the segments inside the pore tend to gather in big clusters that cannot move at all, as is seen in Figure 8. What is also interesting, for the conical pores with $\phi_{trans}>2$, no CLBs that are stuck in the pore have been observed clearly, suggesting that the relation between $\Delta_{min}$ and $\phi_{trans}$ plays an important role in the described phenomenon.

Now we present models for the second of the Moyal distribution parameters: the standard deviation σ. However, in this case, it turns out that it is better to model the behavior of log(σ)—so we obtain the so-called log-linear models. The adopted model functions are the following:

(7) $$\hat{\log(\sigma )}=d_{0}+d_{1}\phi_{cis}+d_{2}\phi_{trans}+d_{3}\frac{\phi_{cis}}{\phi_{trans}}$$

The estimates $d_{i},\;i=0,1,2,3$ of the regression coefficients as well as the model characteristics are provided in Table 2.

The characteristics presented in the last three columns of this table indicate the good quality of the developed models. It can also be seen again that the *p* values confirm our observation that was discussed above: the longer the pore, the greater the impact of the ratio of the diameters on the translocation–time distribution.

## 4. Final Remarks

By employing a sequential algorithm, we investigated the translocations of CLBs through conical pores in membranes whose thicknesses were comparable with the CLB lengths. Our simulations were focused on the analysis of the impact of the pore geometrical parameters on the translocation process. As a result of the analysis of the experimental results, we identified the Moyal probability distribution as a good probabilistic model for the translocation time in all considered setups. Next, we developed regression models that described the relationship between the parameters of the distribution and the geometric parameters of the conical pore. It appeared that the only model component that reflected the interaction between both diameters was the ratio $\frac{\phi_{cis}}{\phi_{trans}}$. Moreover, these models showed that this interaction is significant only for membranes that are thick enough. One of our future research tasks will be a more in-depth simulation study of this interesting issue and, possibly, the detection of these crucial relationships (between geometric parameters of the pore and of the CLBs) that result in the segments becoming clustered rapidly enough to significantly slow down or even stop the translocation process.

We are aware that our probabilistic approach neglects the biochemical characteristics of bio-molecules which, in turn, are included in algorithms relying on large-scale simulations. Our minimal model, however, is sufficiently efficient and effective to mimic a variety of chain-like body translocation phenomena from the mechanical point of view.

## Figures and Tables

**Table 1:** Estimated regression models (6) for selected pore lengths *L*.

Pore Length	$b_{0}$	$b_{1}$	$b_{2}$	$b_{3}$	$R^{2}$	MSE	CoV
L=10	27637.8	−522.2	−687.7	−614.9	0.837	1196.3	0.064
*p*-values:	0.0000	0.0058	0.0002	0.3951			
L=25	30,857.6	−791.3	−589.9	204.0	0.875	1143.9	0.051
*p*-values:	0.0000	0.0001	0.0005	0.7656			
L=50	39,421.5	−1271.9	−567.7	1566.3	0.888	1417.9	0.048
*p* values:	0.0000	0.0000	0.0043	0.0787			
L=75	52,573.9	−1843.9	−787.6	3123.7	0.943	1392.3	0.035
*p* values:	0.0000	0.0000	0.0002	0.0015			
L=100	70,436.7	−2700.8	−1266.1	4632.9	0.922	2502.7	0.049
*p* values:	0.0000	0.0000	0.0006	0.0062			

**Table 2:** Estimated regression models (7) for selected pore lengths *L*.

Pore Length	$d_{0}$	$d_{1}$	$d_{2}$	$d_{3}$	$R^{2}$	MSE	CoV
L=10	8.9963	−0.08894	−0.09771	−0.06807	0.874	0.158	0.020
*p* values:	0.0000	0.0008	0.0001	0.4751			
L=25	9.1615	−0.1368	−0.0535	0.1113	0.932	0.115	0.014
*p* values:	0.0000	0.0000	0.0014	0.1213			
L=50	9.1120	−0.1946	0.0060	0.4452	0.857	0.178	0.021
*p* values:	0.0000	0.0000	0.7816	0.0006			
L=75	9.6473	−0.2338	−0.0083	0.5692	0.860	0.220	0.025
*p* values:	0.0000	0.0000	0.7575	0.0004			
L=100	8.9997	−0.3043	0.0870	1.1313	0.766	0.342	0.037
*p* values:	0.0000	0.0000	0.0051	0.0000			

## Data Availability

Not applicable.

## Supplementary Materials

The following supporting information can be downloaded at: https://www.mdpi.com/article/10.3390/membranes12020138/s1, Sequential Algorithm Description.
