# Supplementary material for: Simulation Study of Chain-like Body Translocation through Conical Pores in Thick Membranes
Source: Membranes (Basel). 2022 Jan 24;12(2):138. doi: 10.3390/membranes12020138 (PMC8878698; doi:10.3390/membranes12020138)
Supplement: Supplementary file 1 [file membranes-12-00138-s001.zip › membranes-1510334-supplementary.pdf]

Supplementary Material
Sequential Algorithm Description

Zbigniew Domański   Andrzej Z. Grzybowski

Inspired by the notion of a strategy defined within the theory of games we present an algorithm designed to study a chain-like body (CLB) propagation through an ensemble of its conformations. In the game theory the idea of a strategy makes it possible to reduce any sequence of possible decisions made by a player during the course of the game to exactly one choice of *a strategy*. What is important in the definition of a strategy is the observation that when each decision is made (in the sequence) then some *a priori* possible situations become no longer accessible. Consequently, when a given strategy is adopted a player does not have to consider all *a priori* possible game "configurations" but only those which can be accessed with the help of this strategy. We adopt the same idea in our algorithm. From a given instant conformation the CLB skips to its another allowable conformation according to a strategy adopted by nature. Within this algorithm a move between two consecutive conformations is built up from a set of virtual steps related to elements of the chain.

In this Supplementary Material we introduce the algorithm in a formal fashion. For this purpose, we need to introduce some terminology.

An abstract $2D$ CLB *position* is a finite sequence $\mathbf{c} = \{c_1, c_2, \ldots, c_n\}$ of points $c_i = (x_i, y_i)$ such that the distance (Dis) between any pair of its successive elements is bounded by given limits L_{min}, L_{max}, i.e. $L_{min} \leq Dis(c_i, c_{i+1}) \leq L_{max}$. The limit L_{max} reflects some constraints as, e.g., constraints imposed by interactions between segments. For example, the Finite Extensible Non-Linear Elastic (FENE) potential includes the maximum bond extension, i.e., the maximal allowed separation between two consecutive monomers equals to L_{max}.

The elements of the sequence $\mathbf{c}$ are called *segments' positions* (shortly *segments*) of the CLB.

Assumption 1 (discretization of the motion space): The CLB moves along the integer lattice nodes, i.e. coordinates of each segment (x_i, y_i) are integer.

It is common in the literature to distinguish between studies conducted within continuous description of polymer-like bodies and these mapped onto a lattice with a coarse grained view on the CLB structure. Both sorts of description are related by some relevant functional-integral limits and the resulting continuous models yield similar characteristics as the corresponding discrete models. Discrete models themselves may depend on continuous space variables or they are formulated with use of variables defined on the countable sets of points, as e.g. on a lattice nodes. However, even in the discrete version to

achieve better approximation of the continuous motion space, one may assume that the abstract unit of the length (distance) is equivalent to a given number of grid sides. Obviously, the greater is the number of grid sides per unit (GSPU), the better approximation of the continuous space. On the other hand greater values of GSPU lead to more computationally demanding simulations.

Any pair (c_i, c_{i+1}) of consecutive segments is called an (i–th) *bond*, $i = 1, 2, \ldots, n-1$. Number n is called the *length* of the CLB. The *structure* of the CLB is defined by the relative mutual positions and orientations of all segments and bonds.

In the description of the algorithm we distinguish *steps* and *moves*. A *step* is made by a single segment of the CLB, carrying it from one lattice node to another. The steps made by segments may be influenced by various external *laws*. For example there may exist a variety of driving forces generated externally. Such outer rules may favor certain lengths or directions of a step. In our algorithm the impact of such external laws can be incorporated by a specific probability distribution π defined on a set of nodes that can be reached by a segment in a single step. This set will be called *one step reachable nodes* (OSRN) and consists of nodes (say b), which for a given segment c satisfy the following condition: $Dis\,(c, b) \leq R_{max}$. The parameter R_{max} equals assumed maximal length of a step made by a segment in a case where no other (internal) forces and/or restrictions are present. This probability distribution determines the more and the less probable directions and/or lengths of the steps. Such a *one step probability distribution* (OS p.d.) may also depend on the segment's coordinates (i.e. its position in a motion space).

Due to assumed properties of the environment and as a result of assumed features of the CLB itself, for any given segment among its OSRN there may exist *actually forbidden nodes* (AFN). For example, one of such restrictions is the upper limit for the distance between subsequent segments. Such a restriction assures the continuity of the CLB. Another example of this kind of restrictions may be a requirement that in a given node, at most a given number of segments can be placed (e.g. the self-avoidance restriction). Yet another example is the existence of different objects that already occupy some nodes, such as a cell's membrane or any other kind of obstacles.

For any given segment the subtraction of the sets OSRN and AFN will be called a set of *actually accessible nodes* (AAN): AAN = OSRN $\setminus$ AFN. The OS p.d. truncated to the set AAN will be called *actual step probability distribution* (AS p.d.)

Let us illustrate the introduced notions in exemplary graphs. In these graphs we assume GSPU = 2 and the following values of remining parameters: $L_{min} = 1$, $L_{max} = 3$ and $R_{max} = 4$ (i.e. correspondingly 2, 6 and 8 grid sides).

The algorithm does not assume any specific form of the distance function $Dis(\cdot, \cdot)$. It can be implemented with any metric which is suitable for a description of a given physical process. In our examples the distance between segments $c = (x, y)$ and $c' = (x', y')$ is defined by a metric:

$$Dis(c, c') = \max\{\mid x' - x \mid, \mid y' - y \mid\} \tag{1}$$

In the topology induced by the metric (1) any sphere with the center c and radius r (in terms of grid sides) consists of all nodes lying in a square with the same center and with sides parallel to the axes having a length $2r+1$. Under our assumptions about the parameters' values, the set OSRN connected with the segment c indicated by the gray bullet in Fig. 1A is the union of all nodes indicated by the empty circles. Consequently, the OS p.d. should be defined on this OSRN set. If all directions and lengths of steps are equally possible, then the OS p.d. is the uniform one. For simplicity of the illustration let us assume the latter case in our example.

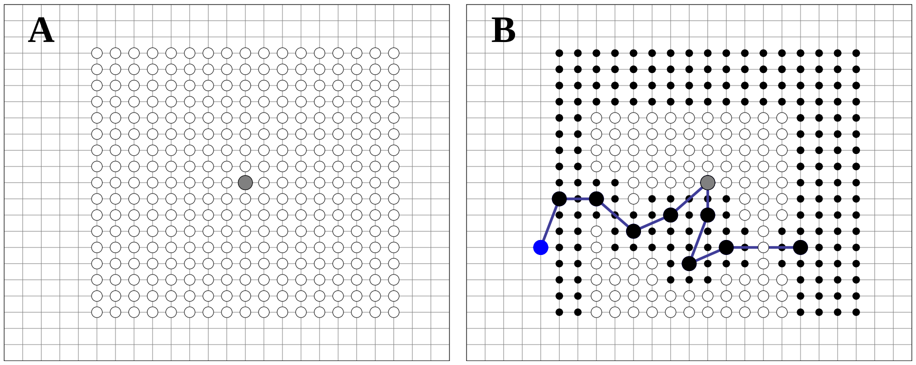

Figure 1: Exemplary segment (indicated by the gray bullet) and its set of OSRN (empty circles in graph A). Sets of AFN (black dots and bullets) and AAN (empty circles) related to the indicated by gray bullet segment are presented in graph B. The assumed values of the problem parameters: $L_{min} = 1$, $L_{max} = 3$, $R_{max} = 4$ and GSPU = 2

To illustrate the notions of the sets AFN and AAN let us assume that the segment c (gray bullet) belongs to the CLB that is indicated by one blue and eight black bullets connected by the blue bonds in Fig. 1B. In our case the only restrictions result from the values of parameters connected with the assumed elasticity properties of the CLB, i.e., the minimal and maximal distance between successive segments (no other bodies occupy the neighbouring nodes). Thus the set AFN consists of the nodes indicated by black dots and bullets while the set of AAN is indicated by the empty circles.

By making a step towards its new position c_i' the segment c_i may *create a tension* in the CLB structure. It is assumed that the tension is big enough to drag neighbouring segment if $Dis\left(c_{i-1}, c_i^{'}\right) > T_d$ and/or $Dis\left(c_i^{'}, c_{i+1}\right) > T_d$, where T_d is a tension parameter. A *move* of the CLB is a sequence of consecutive segments' steps, that finally leads to the relaxation of the structure's tension created by the initial step. In other words, the move reflects the tension propagation and it is finished as soon as there is no tension in the structure. Obviously sometimes the move may consist of a single step only.

Assumption 2 (sequentialization of the CLB move): Every move of the CLB is initialized by only one segment. Then each move of the CLB can be sequentialized into a sequence of *steps*.

The segment that initializes a move of the CLB will be called a *first to step* (FTS) segment. In the algorithm the choice of the FTS segment is random and realized according a given probability distribution defined on all CLB segments. The distribution will be denoted as FTSC p.d. The FTSC p.d. may model various physical aspects of the CLB, e.g. the constrained motion of a tagged monomer or forced relocation of a polymer capped with specific end monomers.

An example of a move made by the CLB from Fig. 1B is illustrated in Fig. 2. First let us number all segments: $c_1, \ldots, c_{10}$. In Fig. 2 the letter "c" is omitted for clarity of the graphs. Let us also assume that the tension parameter is $T_p = 4$ and let us consider the case where the segment c_6 was carried from its initial position to the new c_6' indicated by the green bullet. Now the distances between the new position c_6' of c_6 and its neigbouring segments c_5 and c_7 are greater than T_p. Thus both latter segments have to make the steps. If their new positions c_5' and c_7' are as indicated by the appropriate green bullets in Fig. 2B then again the tension is created between the pairs of segments c_4, c_5' and c_7', c_8. Consequently, the segments c_4 and c_8 have to also make their steps. However, because their new positions c_4' and c_8' do not create any tension in the CLB, the move is completed. The final new position of the CLB is indicated by blue and green bullets in graph B in Fig. 2. Note that during the run of the simulation all new positions of the segments are chosen according the related AS p.d.

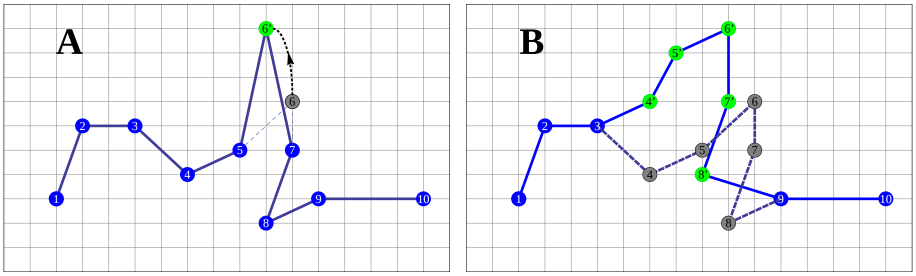

Figure 2: Exemplary move made by a CLB. The assumed values of the problem parameters: $L_{min} = 1$, $L_{max} = 3$, $R_{max} = 4$, $T_d = 2$ and GSPU = 2

A *movement trajectory* is a sequence of consecutive CLB positions stored in matrix $\mathbf{C}$ whose i-th row is interpreted as a CLB position after $i - 1$ moves (at moment i). Thus element C_{ij} denotes the segment j in the CLB position at the moment i.

In many real-world situations, such as biopolymer behaviour inside a living tissue, one should also take into account some additional constraints connected with the biochemical nature of the system. Thus we define additionally the cost connected with the CLB structure. The cost of the CLB structure and its location in the motion space is a function F representing its *fitness* connected

with its conformation and/or other external (e.g. environmental) properties. The lower the cost, the better the fitness of the polymer structure and position.

Assumption 3 (acceptance of new CLB position): The new position of the CLB is accepted with a probability depending on its cost.

The above three assumptions and ideas are implemented in the following sequential algorithm for the CLB movement simulation:

Step 0. (*Initialization*) Set the initial (current) CLB position $\mathbf{c}_{curr}$ and evaluate its *current* cost function value F_{curr}.

Step 1. (*FTM segment selection and computation of its AS p.d.*) According the given FTMCD, select FTM segment $c_{curr,f}, f \in \{1,\ldots,n\}$. For the given segment c_{curr} **determine** sets OSRN, AFN and AAN as well as the AS p.d..

Step 2. (*Step choice for FTM segment)* According to computed AS p.d. randomly select one node out of the set AAN for the next position of the segment c_{curr}.

Step 3. (*Successive steps of remaining segments)* To obtain a new CLB position $\mathbf{c}_{new}$ sequentially choose segments $c_{new,i}, i = f-1, f-2, \ldots$, **determine** their sets SRN, AFN, and AAN as well as the ASPD. Then according to computed AS p.d. randomly draw their subsequent new positions $c_{new,i}$. This process is terminated for the first k,$f-1 \geq k \geq 1$ for which the following condition holds:$d\left(c_{curr,k}, c_{new,k+1}\right) \leq T_d$. If $k > 1$ then $c_{new,i} = c_{curr,i}$ for $i = 1, \ldots k$. Next sequentially choose segments $c_{new,i}, i = f+1, f+2, \ldots$, **determine** their sets SRN, AFN, and AAN as well as the related AS p.d. Then according to computed AS p.d. draw randomly their subsequent new positions $c_{new,i}$. This process is terminated for the first k,$f+1 \leq k \leq n$ for which the following condition holds:$d\left(c_{curr,k}, c_{new,k-1}\right) \leq T_d$. If $k < n$ then, for $i = k, \ldots n$ assume $c_{new,i} = c_{curr,i}$.

Step 4. (*Acceptance of new position)* Compute the cost of the new CLB position F_{new}. If $F_{new} < F_{curr}$ accept $\mathbf{c}_{new}$. If $F_{new} \geq F_{curr}$, accept $\mathbf{c}_{new}$ only if random variable U having a uniform probability distribution on interval $[0,1]$ satisfies $U \leq \psi\left(F_{\text{new}} - F_{\text{curr}}\right)$, with ψ being a given *nonincreasing* function. If $\mathbf{c}_{new}$ is accepted then $\mathbf{c}_{\text{curr}}$ is replaced by $\mathbf{c}_{\text{new}}$; else $\mathbf{c}_{\text{curr}}$ remains as is.

Step 5. Terminate the algorithm if the stopping criterion is met; otherwise return to Step 1.

Step 6. Return the final position of the chain, its cost and required statistics connected with the simulated movement trajectory.

The nonincreasing function that appears in Step 4 of the above algorithm represents the attitude of nature towards the acceptance of worse states. If nature accepts all states, one may assume that $\psi \equiv 1$. Otherwise, similarly as in the famous Metropolis algorithm we propose the use of function $\psi\left(z\right) = \exp\left[-z/T\right]$, where $T > 0$ is a parameter which can be additionally subject to change during the movement process.
